# COVID-19 vaccine hesitancy in an ethnically diverse community: descriptive findings from the Born in Bradford study

**DOI:** 10.12688/wellcomeopenres.16576.2

**Published:** 2021-08-16

**Authors:** Josie Dickerson, Bridget Lockyer, Rachael H. Moss, Charlotte Endacott, Brian Kelly, Sally Bridges, Kirsty L. Crossley, Maria Bryant, Trevor A. Sheldon, John Wright, Kate E. Pickett, Rosemary R.C. McEachan, Bradford Institute for Health Research COVID-19 Scientific Advisory Group

**Affiliations:** 1 Bradford Institute for Health Research, Bradford Teaching Hospitals NHS Foundation Trust, Bradford, BD9 6RJ, UK; 2 Department of Health Sciences, University of York, York, UK; 3 The Hull York Medical School, University of York, York, UK; 4 Institute of Population Health Sciences, Barts and The London School of Medicine and Dentistry, London, UK; 5 Faculty of Life Sciences and Health Studies, University of Bradford, Bradford, UK

**Keywords:** Covid-19, vaccine hesitancy, trust, health beliefs, poverty, health inequalities, ethnicity, social determinants of health, cohorts, Born in Bradford

## Abstract

**Background**: The roll out of coronavirus disease 2019 (COVID-19) vaccines are underway in the UK, and ensuring good uptake in vulnerable communities will be critical to reducing hospital admissions and deaths. There is emerging evidence that vaccine hesitancy is higher in ethnic minorities and deprived areas, and that this may be caused by distrust and misinformation in the community. This study aims to understand COVID-19 vaccine hesitancy in an ethnically diverse and deprived population of Bradford through the Born in Bradford (BiB) research programme.

**Methods**: Surveys were sent to parents in BiB who had taken part in a previous Covid-19 survey (n=1727). Cross tabulations explored variation by ethnicity and deprivation. Answers to a question asking the main reason for hesitancy was analysed using thematic analysis.

**Results**: 535 (31%) of those invited between 29 ^th^ October-9 ^th^ December 2020 participated. 48% were White British, 37% Pakistani heritage and 15% from other ethnicities; 46% were from the most deprived quintile of the Index of Multiple Deprivation. 29% of respondents **do** want a vaccine, 10% **do not.** The majority had not thought about it (29%) or were unsure (30%). Vaccine hesitancy differed by ethnicity and deprivation: 43% (95% CIs: 37-54%) of White British and 60% (35-81%) in the least deprived areas **do want** a vaccine, compared to 13% (9-19%) of Pakistani heritage and 20% (15-26%) in the most deprived areas. Reasons for not wanting a vaccine were commonly explained by confusion and distrust which was linked to exposure to misinformation.

**Conclusions**: There is a risk of unequitable roll out of the vaccination programme in the UK with higher vaccine hesitancy in ethnic minorities and those living in deprived areas. There is an urgent need to tackle misinformation that is leading to uncertainty and confusion about the vaccines.

## Introduction

The roll out of the first approved coronavirus disease 2019 (COVID-19) vaccine began on 8 ^th^ December 2020 in the UK. Ensuring good uptake will be critical to reducing hospital admissions and deaths. However, since the beginning of the COVID-19 pandemic there has been what the World Health Organisation has called an ‘infodemic’: an overwhelming amount of information about COVID-19, much of it unchecked and uncontrolled and spread through social media channels ^1^. The relationship between mis-information, distrust and vaccine up-take is multi-factorial and complex ^2^, however, recent qualitative research undertaken during the COVID-19 Pandemic suggested that the overwhelming and contradictory information available about COVID-19 has caused confusion, distrust and distress ^3^. Importantly, this study found that the greater these feelings of confusion and distress, the less positive people were about COVID-19 vaccination. 

A number of research studies in the UK have indicated that 45-64% of the population are likely to accept the COVID-19 vaccines if offered, and that a small proportion (4-9%) say they definitely would not accept a vaccine ^3– 8^. There are indications in these studies that a lack of trust of key organisations and exposure to misinformation increases vaccine hesitancy ^3, 5, 7, 8^. There are also signs that vaccine hesitancy is higher in ethnic minority and deprived communities ^6– 8^; however this evidence comes from studies with a very small proportion of ethnic minority participants (6-9%). Given that ethnic minority and deprived communities have been disproportionately affected by the virus that causes COVID-19 (severe acute respiratory syndrome virus 2; SARS-CoV-2) ^9^, it is critical that vaccine hesitancy and concerns in these communities are well understood so that vaccine up-take can be enhanced.

The Born in Bradford (BiB) research programme has harnessed existing strong relationships with participants in their ongoing birth cohorts to help understand the impact of COVID-19 on ethnically diverse families, many of whom live in deprived communities. This programme of research uses a mixed methods longitudinal adaptive approach to provide actionable intelligence to local decision makers about how best to minimise health inequalities and aid the City’s recovery ^10^. As part of this programme, longitudinal surveys have been completed with data collection in the first COVID-19 lockdown (April–May 2020) ^11^, and a follow-up survey in October to December 2020. The latter survey included questions about levels of trust in relation to key organisations and vaccination hesitancy. 

This paper reports findings from the second survey of BiB parents, exploring vaccine hesitancy and trust of organisations, by ethnicity and deprivation and aims to provide insights into the reasons why people are uncertain or unwilling to accept the COVID-19 vaccines.

## Methods

### Study design

Quantitative and qualitative analysis of survey questions from the BiB Covid-19 survey (October–December 2020), collected as part of a larger longitudinal Covid-19 research programme ^10^.

### Setting

Located in the North of England, Bradford is the 6th largest city in the UK. The city has a multi-ethnic population of more than 500,000 people, and suffers from high levels of deprivation and some of the worst health and education outcomes in England. Born in Bradford ^12^ ( www.borninbradford.nhs.uk) is an internationally-recognised applied health research programme which aims to find out what keeps families healthy and happy. BiB includes a vast ‘city of research’ infrastructure which includes detailed health and wellbeing information on more than 30,000 Bradfordians enrolled in three birth cohort studies ^3^. Participants for this study came from the

### Study population

Our sample was taken from parents in the BiB Growing Up study who had taken part in recent pre-Covid-19 follow-up ((n=5,154, 2017–2020) and who had completed the first Covid-19 survey (n=1,581, April–June 2020). Parents had an index child aged between 9–13 ^13^.

### Mode of delivery and data collection

Surveys were sent out by post or email, dependent on participants’ preferences. Follow-up by phone was completed 1-3 weeks later and a reminder postcard/email was sent 3-4 weeks after the first contact. For participants with little or no English, surveys were completed in their main language via phone wherever possible.

### Consent

Participants had previously consented to be a part of Born in Bradford and for their research and routine health and education data to be used for research. For this survey, and as approved by the HRA and Bradford/Leeds research ethics committee, verbal consent was taken for questionnaires completed over the phone and logged in the questionnaire database. Implied consent was assumed for all questionnaires completed via post or online.

### Measures

Key questionnaire domains for the survey were co-produced with the Bradford Institute for Health Research COVID-19 Scientific Advisory Group ^14^, and key policy and decision makers within Bradford and communities. Questions were selected or adapted from other relevant questionnaires. The full survey can be viewed here: https://doi.org/10.7910/DVN/Q0SPIQ

**Figure 1.**
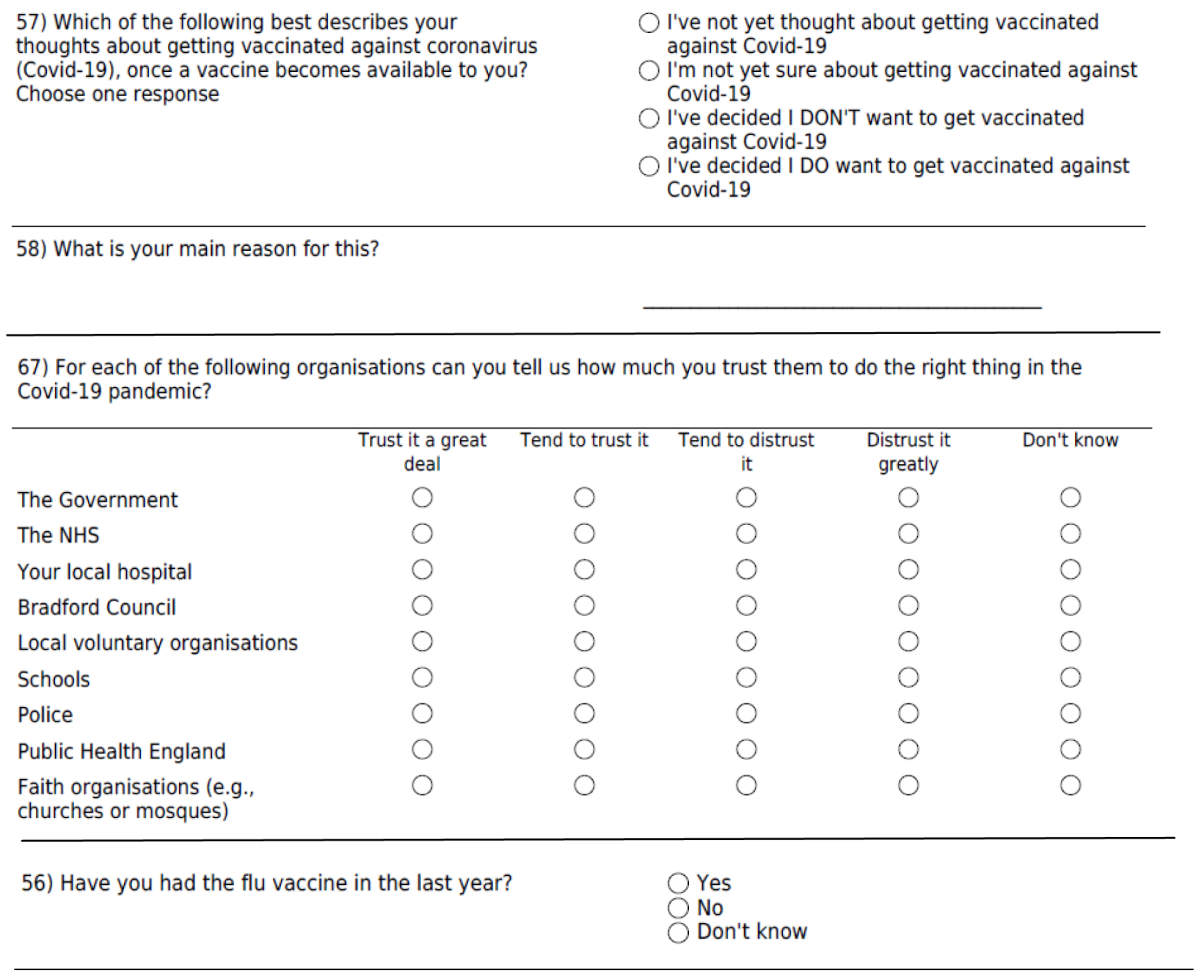
Survey questions on vaccine hesitancy and trust of organisations.

The survey covered key domains on health, wellbeing and economic insecurity as per the first lockdown questionnaire ^11^. We also asked about COVID-19 vaccine hesitancy ^8^, trust of organisations and flu vaccine uptake for this year (winter 2020/21), see Figure 1. Following on from the vaccine hesitancy question, a free text question asked participants “what is your main reason for this [answer]” in order to illuminate the closed question responses.

Ethnicity was captured in self-reported questionnaires administered at baseline recruitment to the cohorts (March 2007 to December 2010) and categorised as ‘White British’, ‘Pakistani Heritage’ and Other (there were small numbers of non-White British, non-Pakistani Heritage parents from multiple ethnic groups). We linked residential address (as at 31st March 2019) to the 2019 Index of Multiple Deprivation (IMD) and composed quintiles of deprivation from least to most deprived ^15^.

### Statistical analysis

Descriptive statistics are presented for each of the survey domains. We used cross tabulations (proportions and 95% confidence intervals) to explore differences in trust and vaccine hesitancy by ethnicity and deprivation. We also explored vaccine hesitancy by trust of different key organisations, and by uptake of the seasonal flu vaccine. All statistical analyses were carried out using Stata 15 ^16^.

### Qualitative analysis

The free text responses to the question asking for the reason for the participants response to the vaccine hesitancy question were explored using thematic analysis ^17^, in order to illuminate the vaccine hesitancy closed question responses. The first 255 responses were analysed by RM and CE, employing an inductive approach where coding and theme development were driven by the content of the responses. A codebook was then developed (by RM, CE and BL) that grouped the responses based on how participants had answered the vaccine hesitancy question to look at key themes for individuals who: a) felt positive about the vaccines; b) were undecided/ had not considered the vaccines; or c) felt negative towards the vaccines. Multiple codes were used within each category to explore and effectively summarise their responses.

The remaining responses were coded by RM and CE alongside frequent discussion with BL to test the strength and validity of the codebook. During this process, thorough and frequent discussion between the researchers took place, allowing adjustments to be made to the original codebook to ensure it was reflective of all responses.

### Ethics

This research was approved by the HRA and Bradford/Leeds research ethics committee (BiB Growing Up study 16/YH/0320).

## Results

Out of a total of 1581 eligible participants, 535 (34%) participated in the study between 29 ^th^ October and 9 ^th^ December 2020.

The mean age of respondents was 42 years (SD 6), with 500 women and 35 men; 234 (48%) were White British, 178 (37%) Pakistani heritage and 74 (15%) from other ethnic groups; 243 (46%) were from the most deprived quintile of IMD. Participants were broadly representative of those who completed the first COVID-19 survey and of those in the entire BiB sample ^10^, but with a drop of ~5% in participation from Pakistani heritage participants and people in the most deprived quintile of the Index of Multiple Deprivation (IMD), see Table 1. There was variation in levels of IMD based on ethnicity, with a higher proportion of Pakistani heritage (70%) and other ethnic groups (58%) living in the lowest quintile of IMD than White Britsh participants (25%) see Table 2.

**Table 1.** Profile of the sample who responded to the Covid-19 surveys compared to the entire BiB cohorts.

	BiB cohort		BiB GU cohort		COVID-19 Survey Phase 1		COVID-19 Survey Phase 2	
Age	Mean	SD	Mean	SD	Mean	SD	Mean	SD
Age as at April 2020	39	6	39	6	40	6	42	6
**Gender**	**N**	**Percentage** **(95% CI)**	**N**	**Percentage** **(95% CI)**	**N**	**Percentage** **(95% CI)**	**N**	**Percentage** **(95% CI)**
Female	12,450	79.1% (78.4%-79.7%)	4617	89.6% (88.7%-90.4%)	1,502	95.0% (93.8%-96.0%)	500	93.5% (91.0%-95.3%)
Male	3297	20.9% (20.3%-21.6%)	537	10.4% (9.6%-11.3%)	79	5.0% (4.0%-6.2%)	35	6.5% (4.7%-9.0%)
Total	15,747	100%	5154	100%	1,581	100%	535	100%
Ethnicity *	**N**	**Percentage** **(95% CI)**	**N**	**Percentage** **(95% CI)**	**N**	**Percentage** **(95% CI)**	**N**	**Percentage** **(95% CI)**
White British	4,636	38.5% (37.6%-39.3%)	1272	28.4% (27.1%-29.8%)	638	43.7% (41.2%-46.3%)	234	48.1% (43.7%-52.6%)
Pakistani heritage	5,366	44.5% (43.6%-45.4%)	2523	56.4% (54.9%-57.8%)	600	41.1% (38.6%-43.6%)	178	36.6% (32.4%-41.0%)
Other	2,055	17.0% (16.4%-17.7%)	682	15.2% (14.2%-16.3%)	222	15.2% (13.5%-17.1%)	74	15.2% (12.3%-18.7%)
Missing	393		140		42		14	
Total	12,450	100%	4617	100%	1502	100%	500	100%
**IMD Quintile**	**N**	**Percentage** **(95% CI)**	**N**	**Percentage** **(95% CI)**	**N**	**Percentage** **(95% CI)**	**N**	**Percentage** **(95% CI)**
1: Most deprived	9366	59.6% (58.8%-60.3%)	3351	65.1% (63.8%-66.4%)	810	51.7% (49.2%-54.1%)	243	45.8% (41.6%-50.0%)
2	3539	22.5% (21.9%-23.2%)	1202	23.3% (22.2%-24.5%)	447	28.5% (26.3%-30.8%)	155	29.2% (25.5%-33.2%)
3	1365	8.7% (8.3%-9.1%)	348	6.8% (6.1%-7.5%)	159	10.1% (8.7%-11.7%)	71	13.4% (10.7%-16.5%)
4	927	5.9% (5.5%-6.3%)	181	3.5% (3.0%-4.1%)	117	7.5% (6.3%-8.9%)	47	8.9% (6.7%-11.6%)
5: Least deprived	527	3.4% (3.1%-3.7%)	68	1.3% (1.0%-1.7%)	35	2.2% (1.6%-3.1%)	15	2.8% (1.7%-4.6%)
Missing			4		13		4	
Total	15724	100%	5154	100%	1581	100%	535	100%

Table shows Mean and Standard Deviation (SD), or Number (N) and 95% Confidence Intervals (95% CI). IMD = Index of Multiple Deprivation. * Ethnicity is shown for women respondents (as male ethnicity was collected using different categories)

**Table 2.** Proportion of participants living in each quintile of the Index of Multiple Deprivation (IMD) by ethnicity.

	White British		Pakistani heritage		Other ethnicity	
IMD quintile	N	Percentage (95% CI)	N	Percentage (95% CI)	N	Percentage (95% CI)
1 (most deprived)	58	25% (20%-31%)	125	70% (63%-77%)	42	58% (46%-68%)
2	69	30% (24%-36%)	49	27% (21%- 34%)	22	30% (21%-42%)
3	60	25% (20%-32%)	3	2% (0%-5%)	5	7% (3%-16%)
4	35	15% (11%-20%)	1	1% (0%-4%)	3	4% (1%-12%)
5 (least deprived)	12	5% (3%-9%)	0		1	1% (0%-9%)
Missing	0		0		1	
Total	234	100%	178	100%	74	100%

### Trust of organisations

Table 3 shows that the most trusted organisations were the NHS (N=432, 89% (95% CIs ), the local hospital (N=415, 85%), and schools (N= 405, 84%). The least trusted were the Government (N= 136, 49%), the local council (N=335, 69%) and faith organisations (N= 326, 67%). There were patterns suggesting differences in trust of organisations by ethnicity, with White British respondents being more likely to trust the NHS and schools, and to be more likely to dis-trust the Government and local council. Pakistani heritage respondents were more likely to trust religious settings. Across all organisations the ‘Other’ ethnic group were more likely to respond ‘don’t know’. However, the variance in responses was low, with small numbers reporting distrust across organisations, making these findings hard to interpret with any certainty (see Table 4). When asked how confident they were that the Government was doing the right thing to stop the spread of COVID-19, 189 (39%) respondents were somewhat or extremely unconfident and 140 (29%) were confident in the Government’s approach, there didn’t appear to be any ethnic differences in responses to this question.

**Table 3.** Trust of organisations, and COVID-19 vaccine hesitancy by levels of trust.

	Total		I’ve not yet thought about it		I’m not yet sure about it		I’ve decided I don’t want it		I’ve decided I do want it		Missing
How much do you trust:	N	Perc.	N	Percentage (95% CI)	N	Percentage (95% CI)	N	Percentage (95% CI)	N	Percentage (95% CI)	N
**The Government**											
Trust it a great deal	49	10%	10	21% (12%-35%)	17	36% (24%-51%)	<5		16	34% (22%-49%)	2
Tend to trust it	205	39%	58	29% (23%-36%)	62	31% (25%-38%)	12	6% (3%-10%)	66	33% (27%-40%)	7
Distrust it	200	37%	46	23% (18%-30%)	62	31% (25%-38%)	30	15% (11%-21%)	60	30% (24%-37%)	2
Don’t know	70	14%	36	51% (40%-63%)	19	27% (18%-39%)	5	7% (3%-16%)	10	14% (8%-25%)	0
**The NHS**											
Trust it a great deal	226	42%	52	24% (19%-30%)	56	26% (20%-32%)	14	6% (4%-11%)	97	44% (38%-51%)	7
Tend to trust it	239	47%	70	30% (24%-36%)	90	38% (32%-45%)	24	10% (7%-15%)	51	22% (17%-27%)	4
Distrust it	27	5%	11	41% (24%-61%)	6	22% (10%-43%)	8	30% (15%-50%)	<5	-	0
Don’t know	34	6%	19	56% (39%-71%)	8	24% (12%-41%)	<5	-	<5	-	0
**The local hospital**											
Trust it a great deal	212	39%	43	21% (16%-27%)	53	26% (20%-32%)	14	7% (4%-11%)	94	46% (39%-53%)	8
Tend to trust it	234	46%	75	32% (27%-39%)	82	35% (30%-42%)	24	10% (7%-15%)	50	22% (17%-27%)	3
Distrust it	33	5%	12	36% (21%-54%)	10	30% (17%-48%)	5	15% (6%-32%)	6	18% (8%-36%)	0
Don’t know	47	9%	22	47% (33%-61%)	15	32% (20%-46%)	7	15% (7%-28%)	<5	6%	0
**Bradford Council**											
Trust it a great deal	76	15%	16	22% (14%-33%)	26	36% (25%-47%)	6	8% (4%-17%)	25	34% (24%-46%)	3
Tend to trust it	282	54%	77	28% (23%-33%)	82	30% (25%-35%)	25	9% (6%-13%)	93	34% (28%-39%)	5
Distrust it	89	17%	23	26% (18%-37%)	29	33% (24%-44%)	13	15% (9%-24%)	22	25% (17%-36%)	2
Don’t know	75	14%	35	47% (36%-58%)	21	28% (19%-39%)	6	8% (4%-17%)	13	17% (10%-28%)	0
**Local voluntary organisations**											
Trust it a great deal	95	19%	24	26% (18%-36%)	26	28% (20%-38%)	10	11% (6%-19%)	32	35%(26%-45%)	3
Tend to trust it	268	52%	71	27% (22%-33%)	85	32% (27%-38%)	26	10% (7%-14%)	81	31% (26%-37%)	5
Distrust it	27	5%	9	33% (18%-54%)	9	33% (18%-54%)	<5	-	5	19% (8%-39%)	0
Don’t know	124	24%	46	37% (29%-46%)	36	29% (22%-38%)	8	7% (3%-12%)	33	27% (20%-35%)	1
**Schools**											
Trust it a great deal	148	29%	35	24% (18%-32%)	42	29% (22%-37%)	14	10% (6%-16%)	53	37% (29%-45%)	4
Tend to trust it	291	55%	83	29% (24%-35%)	89	31% (26%-37%)	25	9% (6%-13%)	88	31% (26%-36%)	6
Distrust it	43	9%	11	26% (15%-41%)	19	44% (30%-59%)	7	16% (8%-31%)	6	14% (6%-28%)	0
Don’t know	41	8%	21	51% (36%-66%)	10	24% (14%-40%)	<5	-	6	15% (7%-29%)	0
**Police**											
Trust it a great deal	130	26%	32	25% (19%-34%)	32	25% (19%-34%)	11	9% (5%-15%)	51	40% (32%-49%)	4
Tend to trust it	268	51%	75	29% (23%-34%)	82	31% (26%-37%)	21	8% (5%-12%)	84	32% (27%-38%)	6
Distrust it	51	9%	16	31% (20%-46%)	21	41% (28%-55%)	9	18% (9%-31%)	5	10% (4%-22%)	0
Don’t know	73	14%	29	40% (29%-51%)	24	33% (23%-44%)	7	10% (5%-19%)	13	18% (11%-28%)	0
**Public Health England**											
Trust it a great deal	141	27%	30	22% (16%-30%)	42	31% (24%-39%)	8	6% (3%-11%)	56	41% (33%-50%)	5
Tend to trust it	235	45%	64	28% (23%-34%)	73	32% (26%-38%)	21	9% (6%-14%)	71	31% (25%-37%)	6
Distrust it	58	11%	17	29% (19%-43%)	19	33% (22%-46%)	11	19% (11%-31%)	11	19% (11%-31%)	0
Don’t know	88	17%	38	43% (33%-54%)	27	31% (22%-41%)	9	10% (5%-19%)	14	16% (10%-25%)	0
**Faith organisations**											
Trust it a great deal	101	19%	28	29% (20%-38%)	34	35% (26%-45%)	12	12% (7%-20%)	24	24% (17%-34%)	3
Tend to trust it	243	47%	74	31% (26%-37%)	73	31% (25%-37%)	17	7% (5%-11%)	73	31% (25%-37%)	6
Distrust it	42	8%	6	14% (6%-29%)	14	33% (21%-49%)	9	21% (11%-37%)	13	31% (19%-47%)	0
Don’t know	134	26%	42	32% (24%-40%)	40	30% (23%-38%)	11	8% (5%-14%)	40	30% (23%-38%)	1

Table shows Number (N), percentage and 95% Confidence Intervals (95% CI). Distrust category contains both ‘distrust it a great deal’ and ‘tend to distrust it’.

**Table 4.** Ethnic differences by Trust in Organisations. (Total N = 486: excludes 35 male respondents where ethnicity coded differently from female respondents, and 14 female respondents with missing ethnicity data;)

	White British (n=234)		Pakistani Heritage (n=178)		Other Ethnicity (n=74)		Total (n=486)	
	N	Perc. (95% CI)	N	Perc. (95% CI)	N	Perc. (95% CI)	N	Perc. (95% CI)
**How confident are you that the Government are doing the right thing to stop the spread of Covid-19?**								
Very confident	8	3% (2%-7%)	16	9% (6%-14%)	4	5% (2%-14%)	28	6% (4%-8%)
Somewhat confident	56	24% (19%-30%)	35	20% (15%-26%)	18	25% (16%-36%)	112	23% (19%-27%)
Neither confident nor	71	30% (25%-37%)	63	36% (29%-43%)	25	34% (24%-46%)	168	33% (29%-37%)
Somewhat unconfident	56	24% (19%-30%)	36	20% (15%-27%)	14	19% (12%-30%)	107	22% (19%-26%)
Extremely unconfident	42	18% (14%-24%)	26	15% (10%-21%)	12	16% (10%-27%)	81	17% (14%-20%)
Missing	1		2		1		4	
**How much do you trust: the Government**								
Trust it a great deal	15	6% (4%-11%)	24	14% (9%-20%)	7	10% (5%-19%)	47	10% (7%-13%)
Tend to trust it	105	45% (39%-52%)	55	32% (25%-39%)	27	38% (27%-49%)	191	39% (35%-44%)
Tend to distrust it	53	23% (18%-29%)	32	18% (13%-25%)	12	17% (10%-27%)	101	20% (17%-24%)
Distrust it greatly	42	18% (14%-24%)	26	15% (10%-21%)	13	18% (11%-29%)	82	17% (14%-21%)
Don’t know	16	7% (4%-11%)	36	21% (15%-28%)	13	18% (11%-29%)	69	14% (11%-17%)
Missing	3		5		2		10	
**How much do you trust: the NHS**								
Trust it a great deal	122	53% (46%-59%)	50	29% (23%-36%)	28	38% (28%-50%)	205	42% (37%-46%)
Tend to trust it	100	43% (37%-50%)	93	54% (46%-61%)	30	41% (30%-53%)	227	47% (42%-51%)
Tend to distrust it	6	3% (1%-6%)	9	5% (3%-10%)	6	8% (4%-17%)	21	4% (3%-7%)
Distrust it greatly	<5	-	<5	-	<5	-	5	1% (0%-2%)
Don’t know	<5	-	17	10% (6%-15%)	9	12% (7%-22%)	34	6% (4%-9%)
Missing	2		5		1		8	
**How much do you trust: the local hospital**								
Trust it a great deal	116	50% (44%-56%)	50	29% (22%-36%)	22	31% (21%-42%)	192	39% (35%-44%)
Tend to trust it	100	43% (37%-50%)	87	50% (43%-57%)	31	43% (32%-55%)	223	46% (41%-50%)
Tend to distrust it	8	3% (2%-7%)	10	6% (3%-10%)	5	7% (3%-16%)	23	5% (3%-7%)
Distrust it greatly	<5		<5		<5			
Don’t know	7	3% (1%-6%)	22	13% (8%-18%)	12	17% (10%-27%)	46	9% (6%-11%)
Missing	2		4		2		8	0
**How much do you trust: Bradford Council**								
Trust it a great deal	32	14% (10%-19%)	33	19% (14%-26%)	7	10% (5%-19%)	72	15% (12%-19%)
Tend to trust it	135	58% (52%-65%)	84	49% (42%-57%)	37	51% (40%-63%)	263	54% (49%-58%)
Tend to distrust it	37	16% (12%-21%)	15	9% (5%-14%)	11	15% (9%-26%)	65	13% (11%-17%)
Distrust it greatly	7	3% (1%-6%)	8	5% (2%-9%)	<5	-		
Don’t know	20	9% (6%-13%)	31	18% (13%-25%)	15	21% (13%-32%)	71	14% (11%-17%)
Missing	3		7		2		12	
**How much do you trust: local voluntary organisations**								
Trust it a great deal	46	20% (15%-26%)	35	21% (15%-28%)	7	10% (5%-20%)	89	19% (16%-23%)
Tend to trust it	132	57% (51%-64%)	76	45% (38%-53%)	33	48% (36%-60%)	247	52% (47%-56%)
Tend to distrust it	7	3% (1%-6%)	10	6% (3%-11%)	6	9% (4%-18%)	24	5% (3%-7%)
Distrust it greatly	<5	-	<5		<5			
Don’t know	44	19% (15%-25%)	46	27% (21%-35%)	23	33% (23%-45%)	118	24% (21%-28%)
Missing	4		10		5		20	
**How much do you trust: Schools**								
Trust it a great deal	73	31% (26%-38%)	50	29% (23%-36%)	14	20% (12%-31%)	139	29% (25%-33%)
Tend to trust it	131	56% (50%-63%)	89	51% (44%-59%)	39	56% (44%-67%)	266	55% (50%-59%)
Tend to distrust it	13	6% (3%-9%)	15	9% (5%-14%)	8	11% (6%-21%)	36	8% (6%-10%)
Distrust it greatly	<5	-	<5	-	<5	-		
Don’t know	11	5% (3%-8%)	17	10% (6%-15%)	9	13% (7%-23%)	41	8% (6%-11%)
Missing	2		5		4		11	
**How much do you trust: Police**								
Trust it a great deal	74	32% (26%-38%)	39	23% (17%-30%)	9	13% (7%-23%)	122	26% (22%-30%)
Tend to trust it	118	51% (45%-57%)	87	51% (43%-58%)	35	49% (38%-61%)	247	51% (46%-55%)
Tend to distrust it	15	6% (4%-11%)	13	8% (4%-13%)	10	14% (8%-24%)	39	8% (6%-11%)
Distrust it greatly	<5	-	6	3% (2%-8%)	<5	-		
Don’t know	23	10% (7%-15%)	27	16% (11%-22%)	17	24% (15%-35%)	73	14% (11%-18%)
Missing	3		6		3		12	
**How much do you trust: Public Health England**								
Trust it a great deal	79	34% (28%-41%)	42	24% (18%-31%)	10	14% (8%-24%)	132	28% (24%-32%)
Tend to trust it	109	47% (41%-54%)	72	41% (34%-49%)	33	46% (35%-58%)	220	45% (41%-50%)
Tend to distrust it	14	6% (4%-10%)	20	11% (8%-17%)	7	10% (5%-19%)	42	9% (6%-12%)
Distrust it greatly	<5	-	<5	-	<5	-		
Don’t know	26	11% (8%-16%)	36	21% (15%-27%)	18	25% (17%-37%)	85	17% (14%-20%)
Missing	4		4		3		12	
**How much do you trust: Faith organisations**								
Trust it a great deal	36	16% (11%-21%)	47	27% (21%-34%)	12	17% (10%-28%)	97	20% (17%-24%)
Tend to trust it	105	45% (39%-52%)	86	49% (42%-57%)	33	48% (36%-60%)	229	47% (43%-52%)
Tend to distrust it	18	8% (5%-12%)	7	4% (2%-8%)	<5	-	28	6% (4%-8%)
Distrust it greatly	7	3% (1%-6%)	<5	-	<5	-	-	-
Don’t know	65	28% (23%-34%)	33	19% (14%-25%)	21	30% (21%-42%)	125	25% (21%-29%)
Missing	3		4		5		13	

### Vaccine hesitancy

Table 5 shows that overall, 154 (29%, 95% CIs: 26-34%) of respondents stated that they **would** want a COVID-19 vaccine, and 53 (10%, 8-13%) said that they **would not** want a vaccine. Most stated they had not thought about it (N= 154; 29%, 26-34%) or were not sure about it yet (N=161; 32%, 27-35%).

**Table 5.** Covid-19 vaccination hesitancy by sociodemographics and flu uptake.

	I’ve not yet thought about it		I’m not yet sure about it		I’ve decided I don’t want it		I’ve decided I do want it		Missing	Total
	N	Percentage. (95% CI)	N	Percentage. (95% CI)	N	Percentage. (95% CI)	N	Percentage. (95% CI)	N	N
**Total**										
	**154**	**29%** **(26%-34%)**	**161**	**30%** **(27%-35%)**	**53**	**10%** **(8%-13%)**	**154**	**29%** **(26%-34%)**	**13**	**535**
**By ethnicity**										
White British	44	19% (15%-25%)	66	29% (23%-35%)	21	9% (6%-14%)	99	43% (37%-50%)	4	**234**
Pakistani	71	41% (34%-49%)	63	36% (30%-44%)	17	10% (6%-15%)	22	13% (9%-19%)	5	**178**
Other	23	32% (23%-44%)	22	31% (21%-43%)	11	15% (9%-26%)	15	21% (13%-32%)	3	**74**
**By IMD Quintile**										
1: Most deprived	80	34% (28%-40%)	78	33% (27%-39%)	30	13% (9%-18%)	47	20% (15%-26%)	8	243
2	49	32% (25%-40%)	44	29% (22%-37%)	17	11% (7%-17%)	42	28% (21%-35%)	3	155
3	15	22% (14%-33%)	19	28% (18%-39%)	5	7% (3%-16%)	30	43% (32%-55%)	2	71
4	8	17% (9%-31%)	13	28% (17%-42%)	<5	-	25	53% (39%-67%)	0	47
5: Least deprived	<5	-	5	33% (15%-59%)	<5	-	9	60% (35%-81%)	0	15
**By flu vaccine in the last year?**										
No	123	33% (28%-38%)	128	34% (30%-39%)	42	11% (8%-15%)	80	21% (18%-26%)	5	378
Yes	25	19% (13%-26%)	30	22% (16%-30%)	10	7% (4%-13%)	69	51% (43%-60%)	1	135

Table shows Number (N), percentage and 95% Confidence Intervals (95% CI). IMD = Index of Multiple Deprivation

**Figure 2.**
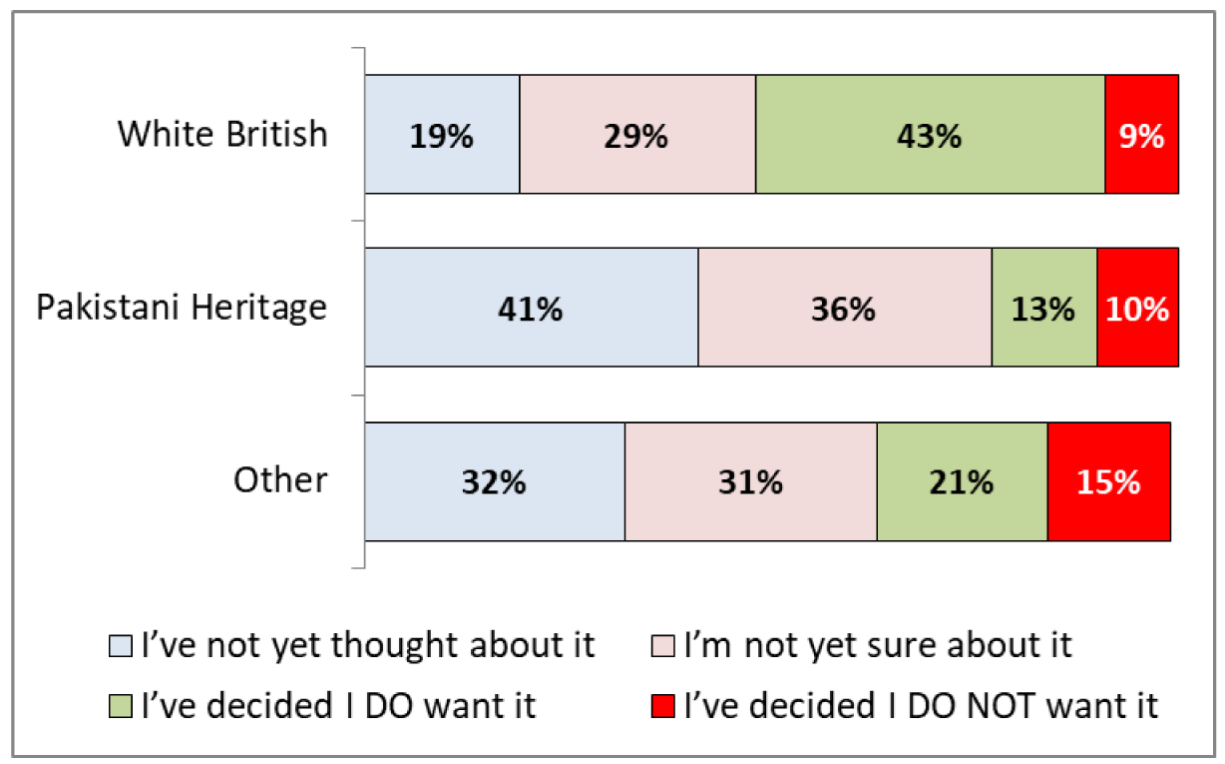
COVID-19 vaccination hesitancy by ethnicity.

Figure 2 shows that there were significant differences in vaccine hesitancy by ethnicity: 43% (95% CIs: 37-54%) of White British respondents said that they **do want** a vaccine compared to only 13% (9-19%) of Pakistani heritage respondents. Pakistani heritage respondents were more likely to be uncertain (36%, 30-44%), or to have not thought about it (41%, 34-49%), rather than stating they would not have a vaccine (10%, 6-15%).

**Figure 3.**
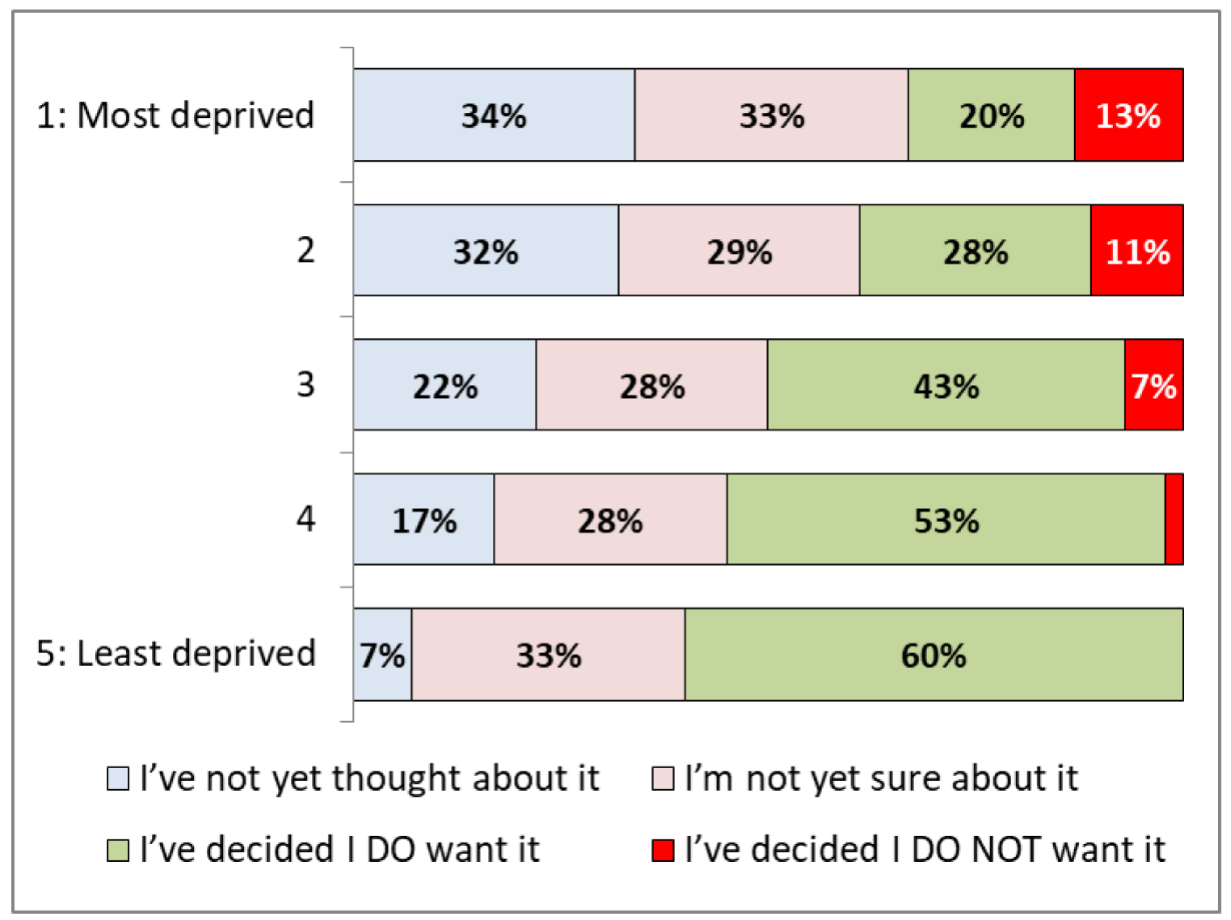
COVID -19 vaccination hesitancy by index of multiple deprivation quintile.

Figure 3 demonstrates significant differences based on levels of deprivation. Of the least deprived quintile of IMD, 60% (35-81%) said that they **do want** a vaccine, compared to 20% (15-26%) in the most deprived quintile.

**Figure 4.**
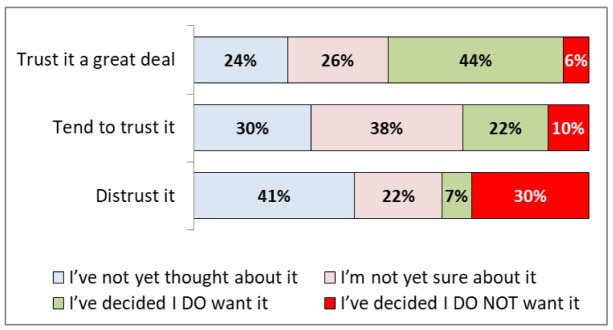
COVID -19 vaccination hesitancy by levels of trust in the NHS.

Figure 4 (see also Table 2) shows that participants who trusted the NHS a great deal were most likely to have decided they want a vaccine (44%, 38-51%), and those that distrusted the NHS were most likely to not want a vaccine (30%, 15-50%). 

**Figure 5.**
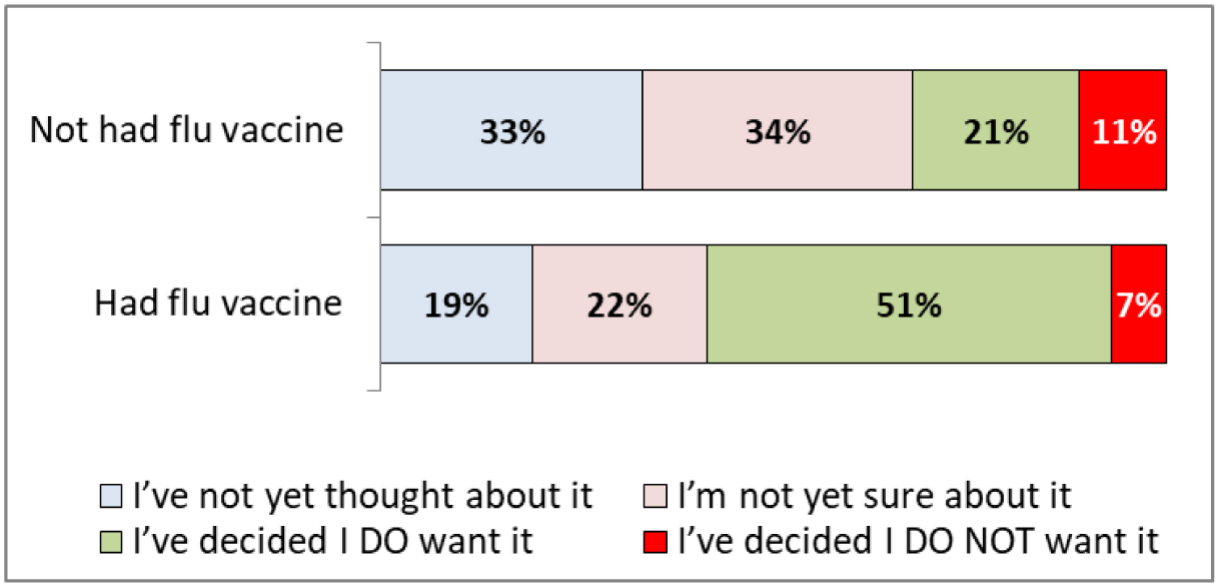
COVID -19 vaccination hesitancy by flu vaccine acceptance.

Figure 5 demonstrates that those that had already had a flu vaccine this year were more likely to want a COVID-19 vaccine (51%, 43-60%).

### Reasons for vaccine Hesitancy response

Of the 535 returned surveys, 64% (n = 343) offered a reason for their response to the question about accepting a vaccine. The main reasons are summarized below based on the response to the vaccine hesitancy questions:

*a) Do not want a vaccine*

Those that had decided **they do not want a vaccine** often stated that there had not been enough research/evidence, it had been ‘rushed through’ and they were concerned about the safety of the vaccines. Their responses were generally stronger and more suspicious in tone than respondents in the other groups, implying and sometimes stating a lack of trust of those that had developed and approved the vaccines:

*I don’t trust the vaccine, it’s been rushed through, side effects etc. just haven't been explored enough*
*Untrustworthy of ingredients*
*Do not trust that the vaccine safety testing will have been rigorous enough, due to being very rushed.*
*I don't trust them*

The lack of trust in the vaccines expressed by some participants’ appeared to be connected to their exposure to misinformation about the COVID-19 virus and vaccines, and these views were very strong in some responses:

*I'm very suspicious of the reasons for the world’s reaction to* COVID-19 *and not sure I can trust what is in the vaccination.*
*Apparently a fix for* Covid *, but at what cost in the future. Most people who get* Covid *will survive it without a vaccine. Vaccinating everyone is a great risk, as no-one had heard of Corona at the beginning of this year. Millions of people walk round with cancer cells, it's interesting none of these companies have ever looked for a vaccine for those!!*

A small number of respondents felt that they did not need a vaccine; either because they were fit and healthy or were taking other precautions, so not at risk:

*I'm healthy and symptom free. Plus I don't feel comfortable having an unknown vaccine*
*Because I'm not in an at risk or vulnerable category.*
*They're not vegan and I don't agree with vaccines. A healthy diet is the best defense.*
*Family is in good health so we don’t need it*

*b) Unsure about having a vaccine*

Those who were **unsure about having a vaccine** expressed concerns about not having enough information to be able to make an informed decision, they were also anxious about not knowing the side effects, the speed with which vaccines had been developed and the safety of the vaccines.:

*Too much of speculations going around that the vaccine is not good so want to know more. Have more info, then will decide.*
*I would like to see the side effects, if any, before committing. I am not an anti-vaccinator, however because it’s new and potentially rushed, would be cautious*
*I'm really anxious about the vaccination because of the speed in which it is being developed. I worry about possible side effects.*

Similar to those who said they did not want the vaccine, these respondents also indicated that exposure to recent and prevalent misinformation had confused them. However these responses tended to be less suspicious than those of participants who did not want a vaccine:

*[Lack of] confidence in fast track development. I know it is unlikely but thalidomide springs to mind for people who took a new drug. That said I do get the flu vac each year and my children are inoculated so I guess I am confused so far.*
*Just unsure about* COVID-19 *in general due to people saying it’s not real etc. I'm confused.*

*c) Not yet thought about having a vaccine*

For those respondents who indicated that **they had not yet thought about having a vaccine**, it is worth noting that the majority of the responses were returned before a vaccine was available to be administered which influenced some of the responses:

*Until a vaccine has been made why ask!*
*Don’t expect vaccines to be ready until mid-2021*
*There’s no imminent vaccine for* COVID-19 *, nothing to think about yet*

It was also apparent from some responses that people were not aware that a number of COVID-19 vaccines were very close to being approved:

*Nothing conclusive has been created.*
*Will be years before vaccine is found*

Similar to the respondents who were uncertain about the vaccine, many responders who said they hadn’t thought about it yet indicated that they were worried about efficacy, safety and potential side effects of the vaccine.

The other key theme that emerged in this group was the need to focus on the present moment and that they did not have the time/space to think about a vaccine right now:

*I am focused on getting through the here and now rather than spending time about what might happen in the future*
*Not thinking about* Covid *anymore fed up of it on TV news everywhere*

## Discussion

This study describes the levels of COVID-19 vaccination hesitancy, and levels of trust of key organisations, in a highly ethnically diverse group of families living in the deprived city of Bradford at the end of 2019. The level of acceptance of vaccination was much lower than found in other studies, with just 29% of respondents being sure they would accept a vaccine, compared to 45-64% found in other studies ^3– 8^. The rates of acceptance in our study were similar to these other studies for our White British participants (43%) and those living in the highest two quintiles of IMD (53–60%). These findings suggest that by failing to include a meaningful proportion of ethnic minority participants, and of those living in deprived areas, previous studies have produced potentially misleading findings.

Born in Bradford is a ‘people powered’ research study; as with other research completed by BiB, the local community were consulted during the Covid-19 lockdowns to understand their concerns and make sure that our research addressed their needs. The Covid-19 survey and recruitment approach were tested through our established research advisory groups and the findings of the study were also shared with these groups to enhance interpretation and ensure useful dissemination back to the community ^10^. We believe that this depth of engagement is what has enabled us to give a voice to seldom heard communities who are particularly vulnerable to the impact of Covid-19 and who have also been more vulnerable to exposure from the ‘infodemic’ of misinformation around Covid-19 and the vaccine.

Our survey also included a free text response asking participants to give the main reason for their vaccine hesitancy response. These responses have helped to illuminate our findings showing that the reasons for not wanting a vaccine included high levels of suspicion or distrust in those that had developed and approved the vaccines, which was often linked to a belief in misinformation about the existence of Covid-19 and the safety and/or the speed with which the vaccine had been developed. Similarly, those who remain uncertain expressed the need for more information, and also some confusion from exposure to misinformation. Those that hadn’t yet thought about vaccination were either focusing on the present moment and didn’t want to think about COVID-19 anymore or were unaware that vaccines were imminent. They also raised similar safety concerns to the undecided group.

These results highlight a much lower level of vaccine acceptability in ethnic minorities, those living in deprived areas and those that distrust the NHS. These findings strengthen the key messages from our recent qualitative work with ethnic minority groups - that there is an urgent need to tackle the overwhelming misinformation about COVID-19 that is leading to uncertainty and confusion about the need for the vaccine, and in the worst cases, a belief that the vaccine should not be accepted ^3^.

The results of this survey have been used to inform local policy through the Bradford District Strategic Coordination Group. A communications strategy has targeted different communities with culturally appropriate messages about the vaccine led by trusted role models and faith leaders. This has included high profile vaccine champions aiming to dispel vaccine myths through multiple media channels and developing a grassroots network of COVID-19 leads to provide neighbourhood advice and support.

We suggest that a wider and similarly carefully targeted response is required to increase vaccine acceptability across the UK, particularly in ethnic minority groups and those living in deprived communities. Most importantly, messaging needs to reassure those who are uncertain or unwilling to think about the vaccines. This messaging needs to be culturally appropriate, provided in non-technical language, and be empathetic to the levels of confusion and distress that people are feeling. This is in direct contrast to the predominantly blanket ‘one size fits all’ messaging from the UK government that has, at times, shown a lack of appreciation of the complexity of people’s living circumstances and of their exposure to misinformation ^18, 19^.

Currently messages regarding Covid-19 and vaccines have been issued by the typo - Government and by local authorities, both of whom have been shown in our survey to be distrusted. There were much stronger levels of trust of the NHS, local hospitals and schools, and in Pakistani heritage communities, also of religious settings. Use of trusted organisations, and of trusted community and faith leaders where appropriate, may help to reassure and encourage those who are currently not willing to accept the vaccine. 

### Strengths and limitations

These findings demonstrate varying levels of trust of key organisations and differential views on vaccine hesitancy based on ethnicity and deprivation. Our study is the first to provide views from a population with a high degree of ethnic diversity and deprivation. Our sample, whilst being diverse, showed variation in deprivation by ethnicity, with a much larger proportion of ethnic minority participants living in the lowest decile of deprivation compared to White British participants. This large overlap in ethnicity and deprivation is important to acknowledge, although one in four White British participants did also live in the lowest quintile. The response rate to this study was also low (31%) and the vast majority of responders were female with an average age of 42 years (which is to be expected as the majority of BiB participants are women recruited during their pregnancy). Non-responders, male participants and different age groups may have different views to those reported here.

Nevertheless our findings do reflect those reported in other studies, with the level of vaccine hesitancy in White British parents matching that found in other studies, as well as a decreased likelihood of vaccine acceptability in those from ethnic minorities and/or living in deprived circumstances.

The approach to allow open text responses to illuminate people’s views on vaccination, also adds strength to this study. The reasons for uncertainty or unwillingness reflect those found in a recent report ^8^.

This study was completed before any of the vaccines had been approved for roll out so there are likely to be some changes in perception now and further exploration of this would be valuable.

The longitudinal nature of the BiB cohorts will allow us to explore change over time and we will continue to follow families throughout the pandemic, adding further value to this research. In addition we have access to routine health data for all participants which will allow us to look at vaccine up-take as data become available throughout 2021.

## Conclusion

Vaccination hesitancy differs based on ethnicity, level of deprivation and trust of key organisations, with those most at risk of serious impact of the virus being the least likely to accept vaccination. Confusion, distrust and distress which was often linked to exposure to misinformation was a main cause of this high vaccine hesitancy. Effective and equitable roll out of the vaccination programme requires careful, empathetic messaging, targeting those whom it will benefit the most, and a multi-organisational approach to address issues of distrust. 

## Data availability

### Underlying data

Scientists are encouraged and able to use BiB data, which are available through a system of managed open access. The steps below describe how to apply for access to BiB data.

- Before you contact BiB, please make sure you have read our Guidance for Collaborators. Our BiB executive review proposals on a monthly basis and we will endeavor to respond to your request as soon as possible. You can find out about the different datasets which are available here. If you are unsure if we have the data that you need please contact a member of the BiB team ( borninbradford@bthft.nhs.uk).
- Once you have formulated your request please complete the ‘Expression of Interest’ form available here and send to borninbradford@bthft.nhs.uk
- If your request is approved we will ask you to sign a collaboration agreement and if your request involves biological samples we will ask you to complete a material transfer agreement.

### Extended data

Harvard Dataverse: Acceptability of Covid-19 vaccination in an ethnically diverse community: descriptive findings from the Born in Bradford study. https://doi.org/10.7910/DVN/Q0SPIQ ^13^

This project contains the following extended data:

- Survey questionnaire
- COVID-19 Code book for free text responses

Data are available under the terms of the Creative Commons Zero "No rights reserved" data waiver (CC0 1.0 Public domain dedication).
