# Supplementary material for: COVID-19 vaccine hesitancy in an ethnically diverse community: descriptive findings from the Born in Bradford study
Source: Wellcome Open Res. 2021 Aug 16;6:23. Originally published 2021 Feb 4. [Version 2] doi: 10.12688/wellcomeopenres.16576.2 (PMC11170065; doi:10.12688/wellcomeopenres.16576.2)
Supplement: Peer review history

# Reviewer report: Reviewer response for version 2

**DOI:** 10.21956/wellcomeopenres.18902.r85248

**Published:** 2024-06-12

**Authors:** Kingsley Kalu

**Affiliations:** 1 Georgia Southern University, Statesboro, Georgia, USA

The authors discussed an important topic, “vaccine hesitancy,” a chronic public health problem that has led to morbidity, mortality, and decreased life expectancy. While the authors try to discuss the association between misinformation and COVID-19 in ethnically diverse groups, there is a need to add some details to provide readers with more clarity. There are a lot of gaps in providing new information in terms of COVID-19 vaccine hesitancy.

 Abstract

- The study design should be stated in the methods section
- The authors indicated n=1727 in the methods section, but 535 was noted in the results section. Please clarify the actual sample size used for analysis.

Introduction:

- The authors stated this sentence in the first part of the introduction: “Importantly, this study found that the more significant these feelings of confusion and distress, the less positive people were about COVID-19 vaccination,” this may be premature to add to the introduction section. It is suggested that statements like this be moved to the discussion section.
- The second section of the introduction should be about vaccine hesitancy: definition, history, and other associated factors. Authors may want to provide more context on this as it relates to the United Kingdom.
- The study’s objectives need to be clearly stated in the last section.

Methods:

- The sample population needs to be differentiated from the actual sample size, and the author's writer should categorically state the sample size for analysis.
- The study design for the manuscript being reviewed should be stated.
- The authors should clarify how the sentence “parents had an index child aged between 9-13” is relevant to this study.

Results:

- Although the authors mentioned using a thematic approach to analyze the qualitative data, the themes were not actually itemized in the report. While examples of respondents’ responses were included, there is a need to categorize these responses under a common theme.

Is the work clearly and accurately presented and does it cite the current literature?

Partly

If applicable, is the statistical analysis and its interpretation appropriate?

Partly

Are all the source data underlying the results available to ensure full reproducibility?

Yes

Is the study design appropriate and is the work technically sound?

Partly

Are the conclusions drawn adequately supported by the results?

Yes

Are sufficient details of methods and analysis provided to allow replication by others?

Partly

Reviewer Expertise:

Vaccine Hesitancy, structural and social determinants of health, zoonotic infections, and health equity

I confirm that I have read this submission and believe that I have an appropriate level of expertise to confirm that it is of an acceptable scientific standard, however I have significant reservations, as outlined above.

# Reviewer report: Reviewer response for version 1

**DOI:** 10.21956/wellcomeopenres.18268.r44273

**Published:** 2021-06-11

**Authors:** Umair Majid

**Affiliations:** 1 Institute of Health Policy, Management and Evaluation, University of Toronto, Toronto, Ontario, ON M5S, Canada

**General Comments**

 Thank you for the opportunity to review your research study on this very important topic. I have taken the chance to review your paper critically and offer helpful suggestions with the purpose of making it a more useful, clear, and relevant read.  

**Abstract**

- I believe that the abstract needs to be a standalone section of the manuscript that provides all essential details on the research study. Some information is missing that should be included in the abstract for clarity:
  - What was the population? Where were they from? How were they selected? This does not need to be long, but the information I see in the abstract is “ethnically diverse and deprived population,” which seems wholly inadequate, and possibly minimizing the uniqueness of the population you chose to target. It also seems that your definition of deprivation and ethnically diverse are intimately tied; White British (ethnicity 1) are “least deprived” while Pakistani (ethnicity 2) are “most deprived.” I think there is a strong need to clarify this in the abstract to avoid any conflation between characteristics.
  - In the same vein, I would also suggest clarifying the first part of the first sentence in the results: 535 of 1727 of X.

**Introduction**

- While you are correct that there is a relationship between greater confusion/distress, and lower views of vaccination, the relationship isn’t as simple as you are portraying. I think it might be okay to keep this as-is, but you must acknowledge that the relationship is complex, and in some cases, the reverse relationship has been found, emphasizing the need to investigate the relationship fully. I discuss this in a recently published paper, but there are hundreds of papers that emphasize complexity of this relationship:
  - Majid, U., Wasim, A., Bakshi, S., & Truong, J. (2020). Knowledge,(mis-) conceptions, risk perception, and behavior change during pandemics: A scoping review of 149 studies. *Public Understanding of Science*, *29*(8), 777-799. ^1^

**Methods**

- In your introduction, you mention that you used a mixed-methods longitudinal design, but in the methods section, you mention that your study design was a survey. Perhaps this paper describes the survey portion that is one of many components of your broader design? In any case, the “study design” section should be used to clarify the relationship between this paper, other papers, and your broader research program.
- Given that some readers may not know about the Born in Bradford Growing Up family cohort, I think it might be beneficial to discuss its characteristics in this paper. I recognize that you probably have another paper that goes into depth about those characteristics but having a brief summary here is essential to make this paper standalone. I also feel that as someone who is not from the UK or the community, I want to know more about Bradford, to help me to understand the surrounding context of the findings. This might include the total population of Bradford, and how the 1727 was determined.
- Since I see that you analyzed “open responses” using thematic analysis, you should describe the content of those open responses in the measures section in a brief sentence, and why this part was included in the survey.
- The qualitative analysis is missing the approach or process you used to compare between different “types” of responses. So, you analyzed positive, negative, and undecided responses separately. How did you compare them?
- This also seems like an analysis of both quantitative and qualitative data. In this case, it is a mixed-methods survey, and there is an opportunity to explain how you have integrated quantitative and qualitative data from the survey.
  - Fetters, M. D., Curry, L. A., & Creswell, J. W. (2013). Achieving integration in mixed methods designs—principles and practices. *Health services research*, *48*(6pt2), 2134-2156. ^2^
  - Clark, V. L. P. (2019). Meaningful integration within mixed methods studies: Identifying why, what, when, and how. *Contemporary Educational Psychology*, *57*, 106-111. ^3^

**Results**

- Demographics:
  - I certainly appreciate the description of participants at the beginning of the results section. I am wondering if you can offer a bit more clarity on how (if at all) ethnicity and deprivation overlap. The current table does not explore this overlap at all, but if there is an overlap in your population, then it needs to be recognized in your analysis. In other words, are White British mostly in “least deprived” areas and Pakistani and other ethnicities in “most deprived” areas?
- Quantitative Findings
  - Can you perhaps elaborate on trust in organizations part across ethnicity, socioeconomic status/deprivation? This is similar to how you have done for the following sections.
- Qualitative Findings:
  - The fifth quote talks about mistrust and distrust, but it follows your sentence on misinformation. I am not sure that you allow for a strong connection between misinformation and mistrust/distrust. The presentation of qualitative research findings requires that you have a good presentation of findings where each quote and your interpretations are intimately linked.
  - Each quote might also benefit if you identified the ethnicity of each person and if they were from a more or less deprived area.
  - You discuss misinformation in two places. I would suggest that you integrate into one area for coherence and conciseness.
    - “These responses also showed participants’ exposure to misinformation…”
    - “Similar to those who said they did not want a vaccine, these respondents also indicate that exposure to recent and prevalent misinformation…”

**Discussion**

- You compare the proportion of participants who are sure why they accept vaccines between your current study and other studies. Can you cite those other studies in this sentence?
- While your discussion is a good start, I think it is somewhat inadequate and superficial for your findings. Discussions are an opportunity for authors to expand, elaborate, and extrapolate their findings. I don’t think you have done that at all. One reason why I think this is because you have only cited a single study in this section. While the implications section is good, it is also superficial since much of this information is not novel and has already been implemented in jurisdictions worldwide. For example, saying that you need a carefully targeted response, from trusted sources, and in non-technical language are the foundations of knowledge translation and public health education with decades of literature. This leads to the following question: what novel implications do you have? Perhaps the novel implications exist in the minutiae details rather than broad concepts that are somewhat intuitive and obvious to researchers in this area. For this reason, I suggest that you take a deeper dive into some of these concepts, really drawing out the novelty and practical implications that make readers or practitioners take as much out as possible from your wonderful work.

Is the work clearly and accurately presented and does it cite the current literature?

Partly

If applicable, is the statistical analysis and its interpretation appropriate?

Partly

Are all the source data underlying the results available to ensure full reproducibility?

Yes

Is the study design appropriate and is the work technically sound?

Yes

Are the conclusions drawn adequately supported by the results?

Yes

Are sufficient details of methods and analysis provided to allow replication by others?

Partly

Reviewer Expertise:

Qualitative and mixed methods research, implementation science, patient engagement, vaccine hesitancy, risk perceptions and behavior change

I confirm that I have read this submission and believe that I have an appropriate level of expertise to confirm that it is of an acceptable scientific standard, however I have significant reservations, as outlined above.

## References

[1] : Knowledge, (mis-)conceptions, risk perception, and behavior change during pandemics: A scoping review of 149 studies. *Public Underst Sci* .29(8) : 10.1177/0963662520963365 777-799 10.1177/0963662520963365 33073717

[2] : Achieving integration in mixed methods designs-principles and practices. *Health Serv Res* .2013;48(6 Pt 2) : 10.1111/1475-6773.12117 2134-56 10.1111/1475-6773.12117 24279835 PMC4097839

[3] : Meaningful integration within mixed methods studies: Identifying why, what, when, and how. *Contemporary Educational Psychology* .2019;57: 10.1016/j.cedpsych.2019.01.007 106-111 10.1016/j.cedpsych.2019.01.007

# Reviewer report: Reviewer response for version 1

**DOI:** 10.21956/wellcomeopenres.18268.r44280

**Published:** 2021-06-07

**Authors:** Richard Shaw

**Affiliations:** 1 Liverpool Head & Neck Centre, Aintree University Hospital, Liverpool, UK

This is a simple questionnaire that addresses a particularly important and current issue i.e. the wide variation of vaccine hesitancy with ethnicity and deprivation.

 In many ways, this data seems urgent to publish as it reflects the current UK disparities in COVID-19 and hospitalisations. 

 The qualitative treatment of justification for vaccine hesitancy data seems reasonable.

 A few concerns:

- Validity  - as return rate was only 31% - accepting no inappropriate stats used and a semi-quantitive analysis but is this return rate valid?
- The survey was carried out in October-December 2020 so precedes the real data and questions about vaccine uptake by a few months. Why is there such a delay between collecting the data and publishing for such a simple MS? Would these answers still be the same, were they reflected in vaccine take up and to what extent?
- By a similar argument, the mean age was 42 years so not an age group selected as a vulnerable group on the whole, or offered vaccine early on the whole. This may reflect the commonest comment "not sure about it yet" and therefore more recent data would firm up these suggestions.

Having stated that, the headline data on strong association with deprivation and that only 13% of Pakistan heritage actively want the vaccine is very important.

Is the work clearly and accurately presented and does it cite the current literature?

Yes

If applicable, is the statistical analysis and its interpretation appropriate?

Yes

Are all the source data underlying the results available to ensure full reproducibility?

Yes

Is the study design appropriate and is the work technically sound?

Yes

Are the conclusions drawn adequately supported by the results?

Yes

Are sufficient details of methods and analysis provided to allow replication by others?

Yes

Reviewer Expertise:

Cancer surgery; COVID19 and healthcare, head and neck cancer

I confirm that I have read this submission and believe that I have an appropriate level of expertise to confirm that it is of an acceptable scientific standard.
